# Metamizole-induced agranulocytosis (MIA): a mini review

**DOI:** 10.1186/s40348-023-00160-8

**Published:** 2023-08-17

**Authors:** Markos K. Tomidis Chatzimanouil, Ines Goppelt, Yvonne Zeissig, Ulrich J. Sachs, Martin W. Laass

**Affiliations:** 1 Department of Pediatrics, Faculty of Medicine and University Hospital Carl Gustav Carus, Technische Universität Dresden, Dresden, Germany; 2 Institute for Clinical Immunology, Transfusion Medicine, and Haemostasis, Justus Liebig University Giessen, Langhansstr 7, 35392 Giessen, Germany

**Keywords:** Metamizole-induced agranulocytosis, Metamizole, Agranulocytosis, Analgesics, Antibodies against granulocytes

## Abstract

Metamizole is an analgesic, antipyretic, and spasmolytic drug in Germany only approved for the treatment of severe pain or high fever that does not respond to other measures. In recent years, an increased use has been described among both adults and children, often against the approved indication. The most important side effect of metamizole is the development of agranulocytosis (neutrophil count < 500/µL). Incidence of metamizole-induced agranulocytosis (MIA) ranges depending on the study from 0.96 cases per million per year to 1:1602 per patient and metamizole prescription. The risk of agranulocytosis in children remains unclear, but is probably lower than in adults. Female gender and older age are associated with higher incidence, reflecting prescription distribution. MIA is dose-independent and risk seems to increase with duration of intake. In patients with past exposure, re-exposure may lead to rapid onset. MIA is believed to be induced either through immunologic or toxic mechanisms. MIA presents with fever, sore throat, fatigue, and mucosal inflammation, up to ulceration. Even in the case of suspected MIA, treatment with metamizole should be immediately paused and an examination of the blood cell count is required. In case of local or systemic infections, empirical therapy with broad-spectrum antibiotics should be administered. G-CSF therapy should be limited to patients with poor prognostic factors. The patient should be monitored closely until the neutrophil count returns to normal. Re-exposure to metamizole must be avoided.

## Introduction

Metamizole (dipyrone) is an analgesic, antipyretic, and spasmolytic drug belonging to the family of pyrazolones [1]. Due to its potentially severe side effects and their unclear incidence, there is controversy regarding its safety, something that has led to varying levels of use, access, and regulatory restrictions across different countries [2]. For example, while metamizole is not distributed in Anglo-American countries (UK, Canada, USA) and in Scandinavia (Finland, Denmark, Sweden), it is freely available in Spain, Russia, Brazil, Mexico, and Israel, while only accessible through prescription in Germany [3]. The main reason causing this controversy is its propensity to induce agranulocytosis, defined as a neutrophil count of less than < 500/µL. The first systematic investigation of metamizole-induced agranulocytosis (MIA) was performed in the 1980s through the International Agranulocytosis and Aplastic Anemia Study (IAAAS), conducted in Europe and Israel. Analgesic use in the week before the onset of illness was compared between 221 cases of agranulocytosis and 1425 hospital controls and showed an up to 24-fold increased risk of agranulocytosis for metamizole users compared to nonusers in the study regions Berlin, Ulm, and Barcelona, with a lower value observed in the other study areas [4]. Following these results, regulatory changes in Germany reclassified the drug from freely accessible to prescription only and restricted its indication [5].

### Indication of metamizole use

Metamizole is currently licensed in Germany for patients older than 3 months, is subject to prescription, and only approved for the treatment of severe pain under certain conditions. This includes acute pain after injuries or surgery, colic and tumor pain, and other pain if other analgesic measures are not suitable. In addition, metamizole is approved for the treatment of high fever that does not respond to other measures. It should not be used for mild or moderate pain or for the treatment of fever if other antipyretics have not been used beforehand and have not shown sufficient effectiveness [1].

### Mechanism of action

Metamizole possesses an analgesic and antipyretic effect, similar to that of aspirin and also equivalent to that of other non-opioid analgesics, although it has significantly weaker anti-inflammatory properties [6]. The exact analgesic mechanism remains unclear. A possible inhibition of both COX-1 and COX-2, along with the inhibition of prostaglandin biosynthesis in the spinal cord, may contribute to its analgesic activity. Metamizole acts as a pro-drug, which is further metabolized in the body. Recently two new metabolites, which appear to have cannabis receptor binding properties, were identified, a mechanism similar to the analgesic mechanism of paracetamol [7]. Regarding its spasmolytic properties, an effect on ATP-dependent potassium channels and cannabinoid receptors is thought to play a role [3, 7].

### Increased use of metamizole

Recently, the drug has re-gained popularity [8]. After an initial decline due to its reclassification, prescriptions in Germany have increased from < 20 million defined daily doses (DDD) in 1990 to 32 million DDD in 2000 to > 140 million in 2012 [5, 9]. Simultaneously, the number of reports of adverse drug reactions (ADR) increased [1] and spontaneous reports of MIA increased from about 10 in 1990 to > 50 in 2012 [10]. Importantly, off-label use of the drug could be identified in about one quarter of the cases (25%) [10]. Another study demonstrated that metamizole was frequently used in the outpatient setting for the treatment of headaches, contrary to the restrictions of use [5]. The exact reasons for this documented increase are unclear, although the generally good tolerability, especially in elderly patients, its low organ toxicity, and the relatively unproblematic use in patients with comorbidities [9, 11], along with the perception that the low-documented incidence of agranulocytosis and ADR in general is not high enough to justify its avoidance [8], are thought to play a major role.

### Use in children

Regarding the use of metamizole in children, information is scarce. Two prospective observational cohort studies in a general pediatric ward with a main focus on the treatment of infectious diseases, the first conducted during an 8-month period from July 1999 to March 2000 and the second during a 3-month period from October to December in 2008, found an increase in metamizole exposure from 4.7% of patients receiving medication in 1999 to 39.2% in 2008 [12]. A follow-up retrospective analysis assessing the use and ADR of metamizole in the years 2015–2020 for the general wards of a department of pediatrics and adolescent medicine of a German tertiary teaching hospital on all inpatient stays revealed a prevalence of 30% in patients receiving drug therapy and 17.3% in all cases, with exposure being highest among adolescents (37.9%) and lowest in newborns (9.9%), proving that metamizole is commonly used and is an important substance in pediatric drug therapy [11]. In Spain, a prospective survey of postoperative treatment in children aged 3–14 years on the first postoperative day showed that metamizole was one of the most frequently used analgesics and another German prospective analysis in eight German tertiary care pediatric oncology centers on pediatric cancer pain management revealed metamizole and paracetamol as the most frequently administered non-opioid treatments in pediatric oncology [7].

### Agranulocytosis as a side effect and its incidence

The most important side effect of treatment with metamizole is the development of agranulocytosis, defined as a drop in neutrophilic granulocytes below < 500µL/µl blood [5, 9, 11]. It should be noted that metamizole-dependent blood count changes, although rarely, also include pancytopenia [9]. Incidence and risk of MIA remain unclear and vary widely: depending on the study, it was 1.5 to 40 times more likely to develop agranulocytosis with metamizole than if the drug was not administered [2, 3]. The IAAAS initially reported an incidence of one case per 1,100,000 user weeks or 6.2 cases per million per year [4]. Further studies in Germany and Spain seemed to support these results [5, 13]. The Berlin Case–Control Surveillance Study [5], a prospective study on adult patients with acute non-chemotherapy-induced agranulocytosis identified by active surveillance in all 51 Berlin hospitals between 2000 and 2010, reported an even lower incidence rate of 0.96 cases per million inhabitants per year, while a retrospective analysis of reports from Switzerland revealed an estimated incidence rate of 0.46–1.63 per million person-days of use [14]. On the other hand, a Swedish study in 1999 calculated an incidence of one case of agranulocytosis at 1439 prescriptions, resulting to a new removal of metamizole from the Swedish market, after having previously been reinstated following the results of IAAAS [7, 15]. This study was criticized due to being a retrospective analysis based on only 8 MIA cases, but recent evidence from Germany seems to confirm these findings, with a recent retrospective analysis from the years 2010–2013 published in 2019 examining new diagnosis of agranulocytosis or neutropenia identified through ICD code, reporting a risk of 1:1602 to develop agranulocytosis and neutropenia per patient and metamizole prescription [15]. However, the calculated risk in this study might be overestimated, as it does not distinguish between neutropenia and agranulocytosis.

### Pediatric incidence

The incidence of MIA in pediatric patients and the risk of agranulocytosis in children remains unclear [16]. While the risk cannot be determined, case reports suggest that it is not negligible, although probably lower than in adults. A retrospective analysis of spontaneous safety reports showed that only 1–2% and 3–6% of documented cases affected age groups 0–9 years and 10–19 years, respectively [14], while in another study 3.7% of patients were younger than 18 years of age [10]. A study assessing postoperative pain therapy in children after a single parenteral use of metamizole in 1177 children could not identify any MIA cases, although the sample size, follow-up, and identification of agranulocytosis were insufficient to detect all episodes [8, 11].

### Risk factors

MIA appears to be more common in females, with multiple studies reporting an up to twofold higher incidence in women than men, although this could reflect the gender distribution of metamizole prescriptions in the examined regions [5, 10, 14, 16]. If female gender is associated with a higher risk of MIA remains unclear. Similarly, increased incidence could be repeatedly reported in association with older age, although again it remains unclear if this is a reflection of higher medication use in the elderly or if older age represents a risk factor for MIA [10, 14, 17]. Increased risk of MIA through viral infections, including COVID-19 infection, similar to increased risk of other ADR through viral infections as previously reported [18], has been suggested but is not yet fully clear. A case series reported a suspected increased risk of metamizole-associated cytopenia in the context of COVID-19 infection [19], while Blaser et al. reported on increased prevalence of hepatitis C virus infection in patients who developed metamizole-associated leucopenia (yet not neutropenia specifically) [20]. Mortality ranged in various reports from 5% up to 23.6% [3, 10, 16] and was significantly elevated with parallel administration of methotrexate. In a retrospective analysis of German spontaneous reports from 1990 to 2012, a concomitant medication with methotrexate was reported in 26.3% of the fatal cases [10], while Blaser et al. found co-treatment with methotrexate in 4 of the 7 fatal cases reported in their study [14]. Three of those four patients with methotrexate therapy received an immunosuppressive low-dose regimen and two of them had received only one single dose of methotrexate. According to the prescribing information, the hematotoxic effect of methotrexate can be increased through parallel intake of metamizole; thus, co-medication of these two drugs should be avoided [3, 9]

Blood count changes due to metamizole intake are thought to be dose-independent [9, 14, 16]. One study reported an average daily dose within the recommended range for all metamizole-associated hematological ADRs [14], while another showed that only in 1.2% of reported cases the recommended maximum dosage was exceeded [10], both arguing against a typical dose-dependent toxicity. In the same study, oral administration was found in around 87% of the fatal outcomes, while the same percentage in all MIA cases was 84.5%, showing that the route of administration probably has minimal influence on the complication rate [10]. If the higher number of oral administrations reflects a higher risk for the cause of MIA than intravenous intake remains unclear, since the application method of metamizole over the period was not documented. In Germany, parenteral administration is only permitted if oral or rectal administration is not possible, as this increases the risk of hypotensive reactions. It appears that the recommendation of primary oral intake is adhered to in adults, contrary to children, where studies revealed parenteral administration of the drug in about 90% of hospitalized patients [11].

The risk of agranulocytosis seems to increase with the duration of intake, especially after a treatment period of over a week, also according to the prescribing information [1, 9]. However, symptoms can also occur if metamizole intake was several days ago and the patient is no longer taking the substance [10]. In sensitized patients due to past exposure, rapid onset of agranulocytosis is possible after re-exposure [9, 10]. Ten days past the last dose no further risk for MIA is to be expected. Poor prognostic factors regarding MIA include age over 65 years old, neutrophil count < 100/μL, concomitant use of methotrexate, and severe clinical infection such as bacteremia, sepsis or shock, deep tissue infections, or serious underlying disease [9, 14, 17].

### Pathomechanism of MIA

While the pathophysiology of MIA is not yet fully clear, it has been suggested to be induced either through immunologic or toxic mechanisms [17, 21]. Most cases of immune-mediated agranulocytoses are thought to be caused by drug-dependent antibodies, meaning antibodies that react only in the presence of the drug or its metabolites, leading to the destruction of neutrophils [17]. Aminopyrine, another pyrazolone derivate that has been discontinued, is believed to act as hapten and induce antibody complexes with neutrophils, which lead to their destruction, while continuous presence of the drug is required [22]. Due to the cross-sensitivity between the two drugs, MIA was suggested to have a similar pathogenesis [7]. This theory has been supported by other studies on metamizole [23], showing that active metabolites of the drug interact with target cells and function as immunogenic haptens, activating T cell responses against granulocytes [10].

Moreover, in the case of metamizole, the drug dependent antibodies appear to only recognize metabolites of the drug and not the native drug itself [17, 21]. Studies showing different levels of acetylation and clearance of active metabolites of metamizole based on gender or age could explain the suspected difference in incidence and risk of MIA in those groups [24]. In recent years, multiple studies of drug induced agranulocytosis, such as on clozapine, sulfasalazine, and carbimazole, revealed genes associated with increased risk either in the HLA region or in other regions involved in immune responses [25].

Contrary to that fact, recent evidence from in vitro studies disagrees with an immune-system driven mechanistic hypothesis for MIA, suggesting a direct toxic effect of the main metamizole metabolite MAA (N-methyl-4-aminoantipyrine) with hemin on granulocyte precursors [26, 27]. In addition, a retrospective observational case–control study investigating genetic associations with MIA at a genome-wide level in the largest patient cohort available to date could not identify significant genome wide associations and no candidate genes suggesting an immune-mediated mechanism were identified. The authors concluded that “these findings thus suggest that the underlying mechanism for MIA may differ from other agranulocytosis-inducing drugs” [28]. Another retrospective study on the same cohort assessing MIA association with HLA regions concluded that “no major HLA risk allele with a strongly increased frequency among patients with MIA or MIN (Metamizole-induced neutropenia) was detected, thus making a T-cell-mediated immune mechanism restricted by a specific HLA allele unlikely” [25].

Perhaps the separation into immunological and cytotoxic agranulocytosis is an oversimplification, and metamizole cannot be clearly allocated to one or the other mechanism [17]. In any case, further studies are needed to fully understand the pathomechanism of MIA and consequently help produce diagnostic tools for clinicians.

### Symptoms

Since MIA can occur at any point during and even after treatment and because multiple case reports showed that the diagnosis of agranulocytosis was often made very late, it is important to raise awareness between both patient and medical professionals regarding its possible clinical presentation. In some cases, metamizole was administered to treat the very neutropenic fever it had caused, often ignoring blood cell counts revealing neutropenia or agranulocytosis [9]. MIA presents with non-specific symptoms such as fever, sore throat, or exhaustion. More typical for MIA is the combination of the above with additional mucosal inflammation, such as aphthous stomatitis, pharyngitis, tonsillitis, or proctitis, all of which can ulcerate as the disease progresses [1, 3, 9]. In around 60% of reported MIA cases, clinical manifestation and complications were documented, including sepsis, tonsillitis, pneumonia, and fever [10].

### Management

**Fig. 1:**
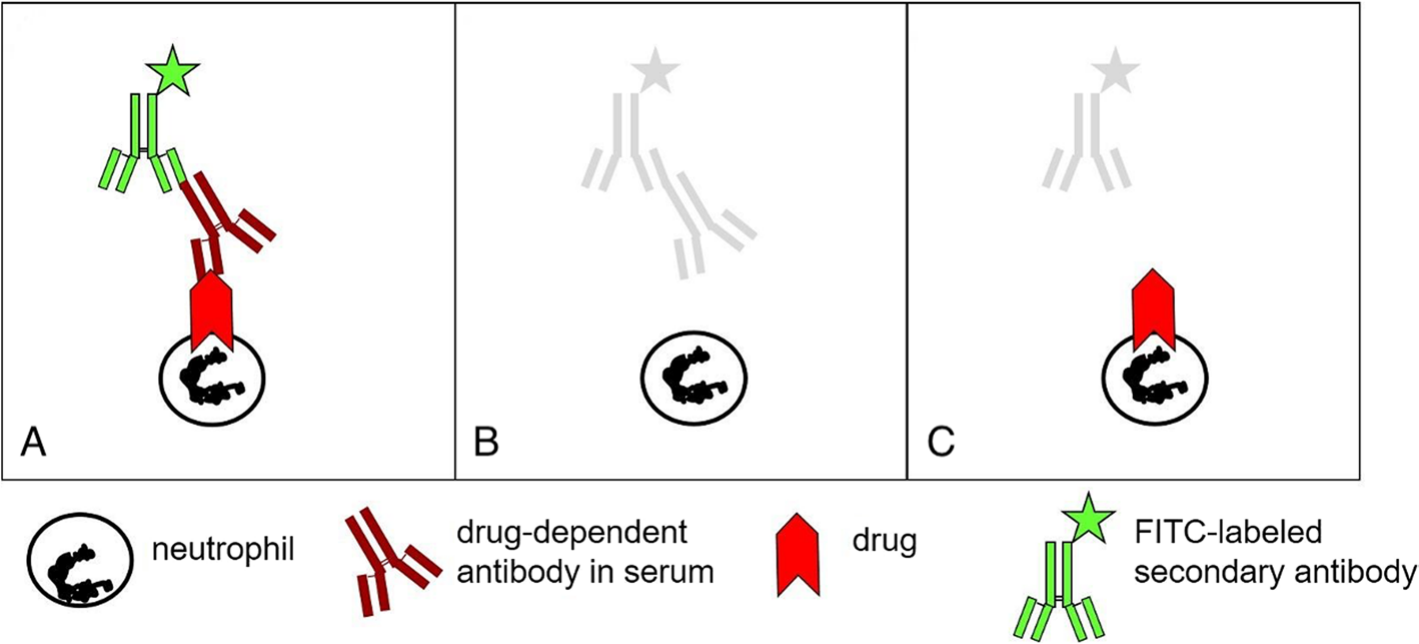
Detection of antibodies against granulocytes by indirect immunofluorescence*. **A** Positive: patient serum and drug. **B** Negative: patient serum without drug. **C** Negative: control serum and drug. *Fixed donor granulocytes are incubated with patient serum in the presence (**A**) or absence (**B**) of the suspected drug. After washing in the presence of the drug, a fluorescence-labeled secondary antibody is used to detect drug-specific antibodies bound to the cell surface. Cells are assessed under a fluorescence microscope against appropriate controls. The test is positive if antibodies are detected in the presence of metamizole or its metabolites [30]

Even in the case of a suspected MIA, treatment with metamizole should be immediately paused and an immediate examination of the blood cell count is required [1, 9, 10]. Detection of metamizole-specific antibodies against granulocytes via indirect immunofluorescence can strengthen diagnosis of MIA (Fig. 1), although the role, the sensitivity, and the specificity of such diagnostic tools on MIA remain unclear, especially considering the unclear pathomechanism of the disease. But agranulocytosis is also a potential side effect of other NSAIDs and paracetamol, albeit less frequently. Thus, until proof of MIA, all other drugs that could cause agranulocytosis should be discontinued. Alternative analgesics that according to current knowledge have no impact on blood cells include opioids like piritramide, hydromorphone, oxycodone, and morphine and can be used in place of NSAID. Non-opioid analgesics can again be administered only if they have been excluded as the causative drug [9].

Due to increased risk of infection and mortality, MIA patients should be accommodated in a single or twin room with ensuite sanitary facilities, but not larger units (three or more patients per room), and basic hygiene measures should be carefully observed [29]. In case of local or systemic infections, the appropriate diagnostic measures should be taken immediately (blood cultures, swab tests, infection parameters) and empirical therapy with broad-spectrum antibiotics should be administered. Many highly effective antibiotic regimens are available and no single empirical therapeutic regimen for the initial treatment can be recommended. Aspects to consider when selecting antibiotic therapy are potential infecting organisms, site of infection, local antibiotic susceptibility patterns, possible organ dysfunction/drug allergy, broad spectrum of activity, and previous antibiotic therapy. Addition of empirical antimycotic therapy should also be considered, as well as surgical intervention [9, 17].

Treatment with granulocyte colony-stimulating factor (G-CSF) has frequently been reported in various case reports of MIA. At the moment, no evidence-based recommendation for use in MIA or other drug-induced agranulocytosis is available. G-CSF has shown to decrease the duration in neutrophil recovery in some studies on non-chemotherapy drug-induced agranulocytosis, while others have shown that the duration of antibiotic therapy, hospitalization, and mortality were also reduced, although none of them was randomized clinical trials or studies specifically on MIA [17]. Even so, potential side effects (musculoskeletal pain, nausea, more severely ARDS, or capillary leak syndrome) should be considered. Administration of this therapy should be limited to patients with poor prognostic factors, as defined above, until further information becomes available [9, 17].

After treatment of MIA, routine blood count checks should be taken until neutrophil count is normalized. Crucial is that the patients are informed to avoid re-exposure to the drug in any case, as this can be fatal. An issue of an allergy pass is recommended [9].

## Conclusion

In conclusion, due to unclear incidence, risk, and absence of clear specific factors associated with MIA, risk minimization for this severe disease can only be achieved through adherence to the indicated use and provision of risk information to patient and health care professionals [10]. Especially due to insufficient proof of safety and general lack of information and evidence in children, we believe that strict attention should be paid to the approved indication when it comes to use of metamizole in pediatric analgesia [6–8]. When administered, it is important to be vigilant for clinical symptoms of agranulocytosis, and intake should be discontinued immediately when MIA is suspected.

Case presentation A 17-year-old girl presented in our emergency department with a 10-day fever of over 40 °C, exhaustion, and sore throat. Antibiotic therapy with penicillin and subsequently cefuroxime had not shown any therapeutic effect. In the clinical examination, we saw pharyngeal erythema, submandibular lymphadenopathy, and white tonsillar exudates. Laboratory test revealed anemia, agranulocytosis, lymphocytopenia, and elevated inflammatory parameters. Extensive search for bacterial or viral pathogens, including blood cultures, throat swab, serological examination for EBV, CMV, Parvo-B19, SARS Cov-2, and HIV remained negative.

**Fig. 2:**
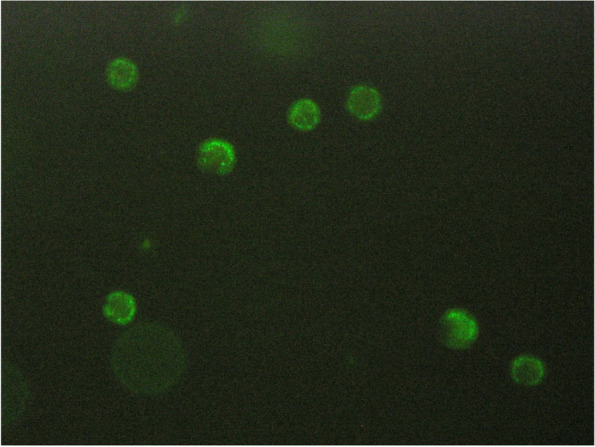
Positive immunofluorescence in our patient

The patient history revealed regular use of metamizole due to menstrual pain. Free anti-neutrophil antibodies were detected in the patient’s serum only in the presence of metamizole, proving MIA (Fig. 2). Treatment of the infections was conducted with intravenous antibiotic therapy with ampicillin/sulbactam, and reverse isolation occurred due to increased infection risk. Under these measures, clinical symptoms resolved rapidly, and under strict avoidance of metamizole, the neutrophil count steadily increased and completely normalized after 2 months.

## Data Availability

Data available on request due to privacy/ethical restrictions.

## Abbreviations

MIA: Metamizole-induced agranulocytosis
G-CSF: Granulocyte colony-stimulating factor
IAAAS: International Agranulocytosis and Aplastic Anemia Study
COX: Cyclooxygenase
ATP: Adenosine triphosphate
DDD: Defined daily doses
ADR: Adverse drug reactions
NSAID: Non-steroidal anti-inflammatory drug
ICD: International Classification of Diseases
HLA: Human leukocyte antigen
MIN: Metamizole-induced neutropenia
